# Identifying Variables That Predict Depression Following the General Lockdown During the COVID-19 Pandemic

**DOI:** 10.3389/fpsyg.2021.680768

**Published:** 2021-05-17

**Authors:** Einav Gozansky, Gal Moscona, Hadas Okon-Singer

**Affiliations:** ^1^Department of Psychology, School of Psychological Sciences, University of Haifa, Haifa, Israel; ^2^The Integrated Brain and Behavior Research Center (IBBR), University of Haifa, Haifa, Israel

**Keywords:** depression, loneliness, COVID-19, intolerance of uncertainty, lockdown, social isolation, emotion evaluation bias

## Abstract

This study aimed to define the psychological markers for future development of depression symptoms following the lockdown caused by the COVID-19 outbreak. Based on previous studies, we focused on loneliness, intolerance of uncertainty and emotion estimation biases as potential predictors of elevated depression levels. During the general lockdown in April 2020, 551 participants reported their psychological health by means of various online questionnaires and an implicit task. Out of these participants, 129 took part in a second phase in June 2020. Subjective loneliness during the lockdown rather than objective isolation was the strongest predictor of symptoms of depression 5 weeks later. Younger age and health related worry also predicted higher non-clinical levels of depression and emotional distress. The results support the diathesis-stress model, which posits that a combination of preexisting vulnerabilities along with stressors such as negative life events are among the factors affecting the development of psychopathology. Moreover, our results correspond with those of previous studies conducted worldwide during the COVID-19 pandemic. Taken together, these findings call for focusing on psychological factors, especially among younger people, to identify individuals at risk for future development of depression and to promote new strategies for prevention.

## Introduction

In January 2020, the World Health Organization (WHO) declared the outbreak of COVID-19 as a public health emergency of international concern. To that point, no effective or available vaccine had been found, leading the WHO emergency committee to declare that the spread of the coronavirus pandemic could be diminished only by early detection, prompt treatment, and isolation (Sohrabi et al., 2020). In response, many countries required citizens to isolate themselves at home or in lockdown facilities to prevent the virus from spreading. In Israel, the government declared a general lockdown on March 17, 2020, excluding only people who work in vital industries. Schools, social frameworks and religious institutions were closed for over a month and many restrictions were placed on travel and social gatherings. This new reality caused social isolation on a massive scale that had never been seen in the 21st century. While the general lockdown was effective in terms of preventing infection, the short-term and long-term psychological impact of this isolation remains unclear and has yet to be dealt with.

The few studies that examined the psychological effects of isolation or lockdown in past pandemics (such as Ebola or SARS) found a high prevalence of symptoms of psychological distress (Brooks et al., 2020). These studies reported high levels of depression (Hawryluck et al., 2004; Mak et al., 2009), stress (Bai et al., 2004), anxiety (Jeong et al., 2016), and even post-traumatic stress symptoms (Hawryluck et al., 2004). Although these studies clearly show negative outcomes as a result of a lockdown period, they were conducted only on small populations exposed to the contagious disease. Hence, large-scale examinations of the effects of lockdown periods are of importance.

Several studies have already demonstrated negative consequences on psychological health emerging from the global COVID-19 outbreak (e.g., see Lee et al., 2020; Lima et al., 2020; Torales et al., 2020). An increase in negative emotions (e.g., anxiety and depression) was found among the Chinese public, while positive emotions and life satisfaction decreased (Huang and Zhao, 2020). In another study conducted in Switzerland, almost 50% of the participants reported a rise in stress levels and 57% reported depressive symptoms following the lockdown period (de Quervain et al., 2020). Moreover, a rise in suicidal tendencies and deaths was associated with the effects of the coronavirus pandemic or lockdown (Buschmann and Tsokos, 2020). The few studies that focused on the Israeli population showed a high prevalence of worry, nervousness and loneliness, and a rise in levels of depression and anxiety during the beginning of COVID-19 (Horesh et al., 2020; Shapiro et al., 2020; Yehudai et al., 2020). These studies highlight loneliness as an important factor affecting the different aspects of current psychological distress, among them stress, financial worries, anxiety and depression levels (Horesh et al., 2020; Palgi et al., 2020; Lipskaya-Velikovsky, 2021). Another alarming study showed that during the lockdown in Israel, the percentage of COVID-19-related substance users increased compared to the period prior to the pandemic outbreak, as manifested in higher reported levels of cigarette, alcohol and cannabis use (Yehudai et al., 2020). Yet the studies conducted so far focused on the psychological effects at the beginning of the COVID-19 outbreak but did not consider possible lasting psychological effects following the lockdown.

The current study aims to fill this gap by defining psychological markers for the future development of psychological symptoms following lockdown. To this end, this study focuses specifically on depression symptoms in the general population. Among clinical psychiatric disorders, Major Depression Disorder (MDD) is one of the most common psychiatric disorders affecting around 264 million patients worldwide (James et al., 2018). Depression is the most severe psychiatric disorder in terms of risk for suicide and one of the three leading causes of non-fatal health loss (Cuijpers et al., 2012; James et al., 2018). It is associated with substantial burden to the patient and causes economic, social, and health impairments (Cuijpers et al., 2012; James et al., 2018).

Recent approaches to the etiology of depression focus on multi-factor models such as the diathesis-stress model, which emphasizes that a combination of preexisting vulnerabilities such as neurobiological factors along with stressors such as negative life events leads to the development of psychopathology (Ingram and Luxton, 2005). With respect to general risk factors for the development of depression, previous examinations have shown the contribution of female gender (Eaton et al., 2008), younger age (Bruce and Hoff, 1994; Kessler et al., 2004; although see Mirowsky and Ross, 1992; Stordal et al., 2003 for evidence of higher depression levels also among individuals above the age of 60), lower employment status (Batterham et al., 2009), stressful life experiences (Kessler, 1997; Assari and Lankarani, 2015), social isolation and loneliness (Bruce and Hoff, 1994; Cacioppo et al., 2010). A meta-analysis by Djernes (2006) further emphasizes the relevance of these factors. The analysis found that the main predictors of depression symptoms in older age are female gender, somatic illness, cognitive impairment, a history of depression, functional impairments, and lack or loss of close social contacts. Furthermore, studies that have examined the psychological effects of isolation or lockdown in past pandemics have pointed to related factors that contribute to psychological distress and depression (Brooks et al., 2020), including personal psychological variables such as worries regarding financial and health status (Hawryluck et al., 2004; Wu et al., 2009), as well as sociodemographic characteristics such as younger age (Taylor et al., 2008), longer duration of isolation (Hawryluck et al., 2004; Mak et al., 2009), and being infected with the contagious disease (Wu et al., 2009).

In the context of pandemic-related isolation, loneliness is a prominent predictor of non-clinical levels of depression. Loneliness is a distressing feeling accompanying the perception that social needs are not being met by the quantity and quality of social relationships (Hawkley and Cacioppo, 2010). Loneliness is often mistakenly referred to as social isolation, defined as a quantifiable social disconnectedness characterized by small social network size and low frequency of social interactions (Cornwell and Waite, 2009). Nevertheless, although loneliness and social isolation are related, they are distinct concepts (Hawkley and Cacioppo, 2010). Studies on the effect of loneliness on quality of life have found that perceived loneliness serves as a risk factor for various physiological and health outcomes. Specifically, loneliness majorly contributes to the development of depression symptoms (Hawkley et al., 2010; Bangee et al., 2014). For example, a 5-year longitudinal study showed a temporal association between perceived loneliness levels and subsequent depression severity, so that loneliness predicted increases in depressive symptoms regardless of other factors such as demographic variables, objective social isolation, stress, dispositional negativity or social support (Cacioppo et al., 2010).

Another timely and relevant predictor of depression symptoms is intolerance of uncertainty (IU). The pandemic outbreak has led to many globally and individually uncertainties on many life aspects. IU is a personal characteristic expressed as a tendency to hold negative beliefs about uncertainty and its implications (Carleton et al., 2012). When faced with ambiguity or uncertainty, individuals high in IU experience elevated stress levels and often use maladaptive coping strategies that may be related to the development and maintenance of depression and anxiety (Carleton et al., 2012). Moreover, an early study have already showed that during the beginning of the COVID-19 outbreak, IU had a significant increasing direct effect on depression, anxiety and stress (Bakioğlu et al., 2020).

Although several recent studies have already examined the consequences of the COVID-19 pandemic in terms of psychological health, most of them used questionnaires and interviews only (Horesh and Brown, 2020), which may be biased by social desirability, self-perception biases or demand characteristics. Unlike previous studies that examined psychological outcomes following COVID-19 using self-report questionnaires, the present research attempted to overcome these problems by using a behavioral measure that indirectly examines psychological distress. Previous research offers evidence for biased emotional face processing in depression (Gur et al., 1992; Surguladze et al., 2005; Bourke et al., 2010; Aue and Okon-Singer, 2020), such that neutral or ambiguous faces are interpreted as negative while happy faces are interpreted as neutral (Gur et al., 1992). In other studies, depressed individuals required significantly greater intensity of emotion to identify happy expressions correctly than did participants with social phobia and healthy participants (Joormann and Gotlib, 2006). Moreover, dysphoric participants showed enhanced memory for angry faces but not for sad, happy or neural faces (Wells et al., 2010). Furthermore, Sanchez et al. (2017) found that depression levels were associated with longer time in disengaging attention from negative faces and that this bias mediated the association between depression levels and self-reported stress recovery, predicting lower recovery from stress. As depressed individuals are more sensitive to signs of interpersonal rejection such as expressions of anger and tend to use excessive reassurance seeking, a growing number of studies have claimed that early presentations of those behaviors represents a vulnerability factor for later development of depression symptoms (Joiner et al., 1999; Davila, 2001; Joiner and Timmons, 2008). Based on these findings, this study used a simple emotional intensity evaluation task to examine the value of emotional evaluation biases in predicting the development of depressive symptoms during the COVID-19 pandemic.

The current short-term study examines the hypothesis that subjective loneliness, IU and emotional evaluation biases predict non-clinical depression levels after a lockdown period. Specifically, we hypothesize that: (*H1*) Higher levels of objective isolation will be positively related to loneliness levels; (*H2*) Non-clinical levels of depression during and after the lockdown period will be predicted by loneliness, IU, age, gender and emotional evaluation biases; (*H3*) Finally, the COVID-19 virus poses many health-related worries, especially for people who are at high health risk. The effects of the pandemic are not limited to health, but also have a major impact on economic aspects, leading to a significant damage to the economy (Abd-alrazaq et al., 2020) as well as elevated worries and financial stress (Abd-alrazaq et al., 2020), suggesting an increase in suicide rates due to lockdown-related economic problems (Kawohl and Nordt, 2020). Therefore, in order to account for the current pandemic and lockdown situation, the prediction models also included economic-related worries and health-related worries as variables predicting non-clinical depression levels.

## Materials and Methods

### Participants

Five hundred fifty-three adults living in Israel (384 female, *age range*: 18–87 years, *M* = 41.02, *SD* = 16.1; see Table 1) completed the first phase of the study. Answering the questionnaire was voluntary basis, means no payment was received for any of the experiment’s phases. The inclusion criterion was being over the age of 18 and full completion of the experiment (i.e., responding to all questionnaires as well as task completion) in no more than 35 minutes. Hence, two participants were excluded from the analysis since they reported being underage, and 215 participants were excluded since they did not complete the whole experiment at the requested time frame. One additional outlier participant was excluded. The ethnic composition of the sample was 77% Jews and 23% Arabs, similar to the ethnic ratio in Israel’s general population. In addition, the sample included participants from all the districts in Israel.

**TABLE 1:** Demographic characteristics of the sample in the first and second phases.

Characteristic	Phase 1	Phase 2
Age (years, *SD*)	41.02 (*16.1*)	43.09 (*17.7*)
Female (%)	69.7	76.7
**Educational level (%)**		
Primary education	0.4	0.8
Secondary education	20.3	18.6
University degree	79.3	80.6
Risk group for COVID-19 infection (%)	24	28.7
Volunteer activity (%)	18.7	17.1
**Employment status (%)**		
Not working at all	41	45
Working from home	45	40.3
Working outside of home	14	14.7
**Household size (%)**		
Living alone	7.4	7.8
With one partner	22.3	27.9
With 2–3 partners	35.2	40.3
With 4–9 partners	34.1	23.3
With 10–20 partners	0.4	0
With 20 partners or more	0.5	0.8
**Close social network size (%)**		
No close friends	1.1	0.8
One close friend	1.8	1.6
2–3 close friends	29.6	27.9
4–9 close friends	49.4	52.7
10–20 close friends	12.2	10.1
20 close friends or more	6.0	7.0
**Number of close social interactions in the last week (%)**		
No interactions with a friend	5.3	4.7
Interactions with one friend	5.8	4.7
Interactions with 2–3 friends	37.6	38.8
Interactions with 4–9 friends	38.8	34.9
Interactions with 10–20 friends	10.7	12.4
Interactions with 20 friends or more	1.8	4.7

Of the 551 participants who fully completed the first phase, 330 participants gave their consent for participation in additional future phase of the experiment (for more information, see Method section). Of them, one hundred twenty-nine participants completed the second phase (98 female, *age range*: 19–80 years, *M* = 43.09, *SD* = 17.6), again on a voluntary basis. The inclusion criterion was similar as phase one.

The samples of both phases did not differ in age range (*t*_(__678__)_ = −1.265, *p* = 0.207) or in female-male ratio (χ^2^_(__1__)_ = 2.512, *p* = 0.113). To further indicate whether the two samples were equivalent in age distribution, non-parametric Levene’s test was performed. This method is suited for examining homogeneity of variance in samples with non-normal distribution or unequal sample sizes (Nordstokke et al., 2011). Non-parametric Levene’s test showed that the variance for age in phase 1 was equal to the variance of age in phase 2 (*F*_(__678_,_1__)_ = 2.833, *p* = 0.093). The experiment was approved by the local Ethics Committee (approval number 141–20).

### Measures

#### Demographic Questions

Participants were asked to report their age, sex, first spoken language, educational level, religious identification, residential area, whether they are at increased risk for COVID-19 complications (i.e., whether they are a part of any risk group for COVID-19 infection), and whether they were diagnosed with COVID-19.

#### Current Stress Questions

Participants were also asked to rate, on a 5-point scale, their current level of worries regarding their health situation (i.e., “how much do you currently worry about your health”) and current level of worries regarding their economic state (i.e., “how much do you worry about your financial state due to the current situation”).

#### Social Isolation

Based on the literature, we formulated several questions that represent different aspects of social isolation and were suitable to the lockdown period (Cornwell and Waite, 2009). The questions reflected the size of the individual’s social network [using two variables: close social network size and number of social interactions in the past week, both rated on a 6-point scale ranging from 0 (20 close friends or more; interactions with 20 friends or more) to 5 (no close friends; no interactions with a friend in the last week)], daily physical contact during the lockdown period (defined by household size during the lockdown, rated on a 6-point scale ranging from 0 (living with 20 partners or more) to 5 (living alone), participation in volunteer activities during the lockdown (e.g., volunteering that was allowed and common at that time, presented as a yes/no question), and marital status. Current employment status was also measured as an indicator of daily contact with other people, as individuals that are currently employed have some contact with co-workers (which varies depending on whether the person is working physically or remotely), as opposed to unemployed individuals. Current employment status was rated on a 3-point scale ranging from 0 (working outside of home) to 2 (not working at all). On each question, a higher score reflects a higher level of isolation. Participants were also asked to indicate whether they were in enforced self-isolation (Yes/No) and when this isolation occurred.

#### The Revised University of California, Los Angeles (R-UCLA) Loneliness Scale

This questionnaire measures subjective feelings of loneliness and social isolation (Russell et al., 1980). For each of 20 statements, participants are asked to use a 4-point scale to indicate how frequently they feel as described in the statement. Total scores range from 20 to 80, with higher scores indicating higher perceived loneliness. We used a Hebrew version translated by Hochdorf (1989). For the Arabic version, we translated and validated the questionnaire using inter-judge reliability, based on Siny et al. (2017). In this study, Cronbach’s alpha was 0.9.

#### The Intolerance of Uncertainty Scale – Short Form (IUS-12)

This questionnaire measures responses to uncertain and ambiguous situations (Carleton et al., 2007) via 12 items that participants rate on a 5-point scale ranging from 1 (not at all characteristic of me) to 5 (entirely characteristic of me). Scores range between 12 and 105. We used a translated IUS-12 Hebrew version (Zerach and Levi-Belz, 2019). For the Arabic version, we translated and validated the questionnaire using inter-judge reliability, based on Siny et al. (2017). In the current sample, Cronbach’s alpha for the IUS-12 was 0.87.

#### The Short Depression, Anxiety and Stress Scale (DASS-21)

This set of three self-report scales is designed to measure the negative emotional states of depression, anxiety, and stress/tension (Lovibond and Lovibond, 1995). The scale contains 21 components that participants rate on a 4-point scale. Scores for depression, anxiety, and stress are calculated by summing the scores for the seven relevant items, with scores ranging from 0 to 21. In our study, we used translated versions: a Hebrew version (translated by Dr. Janine Lurie) and an Arabic version (Moussa et al., 2017). Cronbach’s alpha value for depression was 0.91, for anxiety was 0.77 and for stress was 0.92.

#### Emotional Intensity Evaluation Task

**FIGURE 1:**
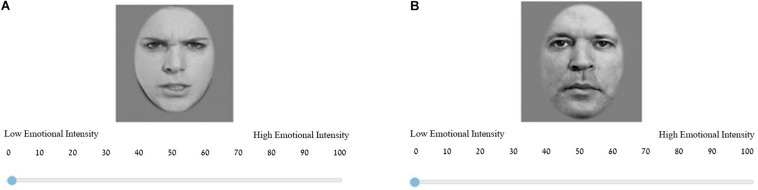
An example of two trials from the experiment. **(A)** A trial containing an angry female face depicting 90% emotional intensity. **(B)** A trial containing a happy male face depicting 10% emotional intensity. The picture stimuli database as well as normative data are based on the stimuli created by Blais et al. (2012), and are available at the following address: http://mapageweb.umontreal.ca/gosselif/STOIC.rar.

Based on previous experiments conducted in our lab (Naor et al., 2018, 2020), we used a set of pictures depicting the faces of ten actor models with different emotional expressions (Blais et al., 2012). The picture stimuli database as well as normative data are available at the following address: http://mapageweb.umontreal.ca/gosselif/STOIC.rar. The emotional expressions were morphed to create a sequential morph of 100 pictures for each character. For detailed information, please see Naor et al. (2018, 2020). In our experiment, six models were used (i.e., three males and three female models), each depicting two emotions (happy and angry) at five levels of intensity (10, 30, 50, 70, and 90%), accounting for a total of 60 exemplars. On the Emotional Intensity Evaluation Task, participants were asked to evaluate the emotional intensity of each exemplar separately, on a scale ranging from 1 (low emotional intensity) to 100 (high emotional intensity) (see Figure 1), without a description or a need to identify the given emotion. Each exemplar was shown once to each participant in random order.

In order to compute *the evaluation bias*, we calculated the deviation from the normative score for a given specific degree of emotional expression for each exemplar (as was rated by another sample from a previous study conducted in our lab, see Naor et al., 2018). For example, judging an exemplar depicting 30% anger as 47% anger represents a positive bias of 17% in anger evaluation for this exemplar. Bias scores can be positive or negative, respectively representing over-evaluation or under-evaluation. The bias was computed and averaged for each emotion and each intensity level across models (i.e., exemplars), yielding ten bias scores (i.e., 5 intensity levels × 2 emotions). Then, five bias scores were used as indicators of the construct of angry emotional evaluation bias (i.e., bias when evaluating angry faces), and five bias scores were used as indicators of the construct of happy emotional evaluation bias (i.e., bias when evaluating happy faces) (Bourke et al., 2010).

### Procedure

The present study was administered through the online study platform Qualtrics (Version April 2020 of Qualtrics, Copyright^©^ 2019 Qualtrics) at two time points. Both phases were available in Hebrew and in Arabic, and participants could choose their preferred language for answering. The first phase was administered to participants in April, during the general lockdown in Israel, where going out of the house was allowed only for essential jobs (e.g., medical doctors), volunteering (such as helping older persons in need), and procurement of medical equipment and food. The second phase was administered 5 weeks later, at the beginning of June, when most of the restrictions in Israel had been lifted. Work and leisure places were gradually opened, and another lockdown was not in sight.

For the first phase, we recruited participants using advertisements posted on social networks. At the beginning of the experiment all participants gave their consent for participating. The first phase began with demographic questions and objective isolation questions, as described above. Levels of economic worry and health-related worries were measured on a 5-point scale ranging from 1 (not stressed at all) to 5 (highly stressed). Then, participants performed the Emotional Intensity Evaluation task and completed the three self-report questionnaires (UCLA, DASS-21, and IUS-12) in a random order. At the end of the experiment, participants were asked to give their consent to participate in future studies. Only participants who gave their agreement were included in the second phase.

In the study’s second phase, participants were asked to complete three questionnaires (DASS-21, PSS, and PTGI) in a random order, and then were asked to report their current levels of economic worry once again. The PSS and PTGI questionnaires are beyond the scope of the current analysis.

### Data Analysis

For each questionnaire, scores that were 3.3 SDs above or below the group average (∼1% of the data) were corrected using score alteration (Osborne and Overbay, 2004). Specifically, outliers were recoded to the highest or lowest remaining score. One participant was excluded due to scores that were 3.3 SDs above the group average on four different questionnaires, which can indicate on abnormal response. For the Emotional Intensity Evaluation task, SDs of the evaluation rates were calculated for each picture separately (specifically, for each character, intensity level and emotion). As for the questionnaire, the same method for outliers detection and score alteration was used (<0.001% of the data).

Principal components analysis (PCA) was used to identify and compute composite scores for the factors underlying social isolation items, using IBM SPSS Statistics, version 25.0 (IBM Corp., Armonk, NY, United States). The social isolation items were transformed to z-scores and entered into an exploratory factor analysis. Kaiser-Meyer-Olkin and Bartlett’s test of sphericity were used to verify sampling adequacy.

The main hypotheses were addressed by constructing structural equation modeling (SEM) models. SEM accounts for multiple accumulative relationships among variables, can handle numerous sources of variance, enables working with latent variables, and makes it possible to test directional hypothesized relationships, thus making it a suitable tool for examining our research questions (Klem, 2000). Maximum-likelihood estimation (ML) was used in the Analysis of Moment Structures (AMOS) module (Version 25.0; Arbuckle, 2017) of the SPSS statistical package to complete the analyses. The weighted least squares (WLS) method (an approach usually preferred for ordinal variables) was not used due to the very large sample size requirements (e.g., 2,000 cases). Moreover, WLS is not preferable to the maximum-likelihood estimation procedure in terms of parameter bias and fit (Olsson et al., 2000). Model fit was assessed using the chi-square goodness of fit statistic, the root-mean-square error of approximation (RMSEA; Browne and Cudeck, 1992), the normed fit index (NFI; Bentler and Bonett, 1980) and the comparative fit index (CFI; Bentler, 1990). We used the criteria suggested by Hu and Bentler (1999) (CFI ≥ 0.95 and RMSEA ≤ 0.06) and the criteria suggested by Marsh and Hau (1996) (NFI ≥ 0.9) as indications of good model-data fit.

#### Phase 1

**FIGURE 2:**
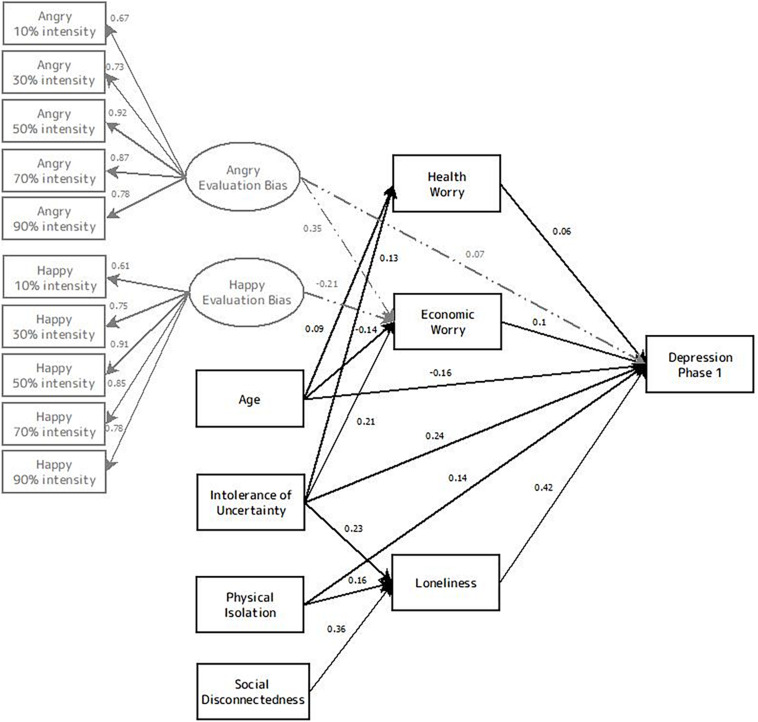
Model of the relationships among age, economic worry, health worry, physical isolation, social disconnectedness, loneliness, intolerance of uncertainty, emotional evaluation bias and symptoms of depression in phase 1. Black lines represent connections in the best fitted model. Gray lines represent non-significant connections that are not included in the best model. Rectangles represent observed variables, and ovals represent unobserved variables. Solid lines represent connections in the best fitted model, and dashed lines represent significant connections that are not included in the best model. Values embedded in unidirectional arrows are standardized regression weights.

The research hypotheses were addressed by constructing a model of the relationships among objective social isolation (i.e., physical isolation and social disconnectedness), loneliness and symptoms of depression. The model also included age, economic worry, health worry, IU and emotional evaluation bias as predicting variables (see Figure 2). A partial hybrid model was constructed, with both unobserved (i.e., latent) and observed (i.e., measured) variables. Both angry and happy emotional evaluation biases were represented as underlying constructs that were measured by multiple observed variables. The variables of age, physical isolation, social disconnectedness and IU were specified as observed variables. The endogenous (i.e., dependent) variable in the model was self-reported symptoms of depression. The constructs of economic worry, health worry and loneliness were considered both as exogenous variables (predictors of depression) and as endogenous variables predicted by the observed variables.

The model was designed based on previous findings regarding variables that predict depression as well as theoretical models for depression development [such as models depicting the relations between loneliness and depression (see Cacioppo et al., 2010) and the diathesis-stress model (see Ingram and Luxton, 2005)], while also considering the inter-connections between the predicting variables themselves. At the first level, we entered variables that were defined as general traits or as generally objective. In the second level of the model, we entered variables that depict psychological outcomes of the situation and are perceived as affected by the variables at level one. Finally, in the third level, our main outcome variable of non-clinical depression levels was entered.

#### Phase 2

The hypotheses were examined using a similar model for phases 1 and 2 together to evaluate the predictive value of physical isolation, social disconnectedness, loneliness as well as age, economic worry, health worry, IU and depression level from phase 1 (e.g., during the lockdown period) on the development and maintenance of symptoms of depression 5 weeks later.

## Results

### Factor Analysis for Social Isolation

Due to the small number of participants who reported being in enforced self-isolation (48 of 551 participants, 8.7%), this item was not entered into the analysis. There was a small difference between the two factors in the Varimax and Oblimin solutions. Thus, we examined both solutions in subsequent analyses before deciding to use a Varimax rotation for the final solution. KMO = 0.52 and Bartlett’s test of sphericity χ^2^_(__15__)_ = 182.65, *p* < 0.001, indicating that the correlation structure is adequate for factor analyses.

The PCA with a cut-off point of 0.40 and the Kaiser’s criterion of eigenvalues greater than 1 (Field, 2009) yielded a two-factor solution as the best fit for the data, accounting for 47.43% of the variance of reported social isolation. The results of this factor analysis are depicted in Table 2. Factor 1 was comprised of three items (e.g., “close social network size,” “volunteering,” and “number of social interactions in the past week”) that explained 25.04% of the variance, with factor loadings from 0.482 to 0.794. This factor represents daily interaction with social networks and thus was labeled “Social Disconnectedness.” Factor 2 consisted of three items (e.g., “employment status,” “marital status,” and “household size during the lockdown”) that explained 22.39% of the variance, with factor loadings from 0.596 to 0.741. This factor represents daily physical interactions and thus was labeled “Physical Isolation.” Composite scores were created for each of the two factors, based on the mean of the items that had their primary loadings on each factor. Higher scores indicate higher levels of isolation on both sub-scales.

**TABLE 2:** Exploratory factor analysis for social isolation.

Item	Component		Dimension
	1	2	
Close social network size	**0.785**	0.200	
Volunteering	**0.482**	−0.051	Social Disconnectedness
Number of social interactions (in the last week)	**0.794**	−0.185	
Employment status	−0.069	**0.606**	
Marital status	0.099	**0.741**	Physical Separation
Household size	−0.069	**0.596**	

*Note. Loadings larger than 0.40 are in bold.*

### Phase 1

#### Psychological State at Phase 1

Table 3 lists the means and SDs for the psychological measures at phase 1. Only one participant reported being diagnosed with COVID-19. Correlations for all measures are given in Table 4.

**TABLE 3:** Means and SDs for the psychological measures at each phase.

Variable	Phase 1		Phase 2	
	*M*	*SD*	*M*	*SD*
Loneliness	35.68	10.22	-	-
Intolerance of Uncertainty	32.63	9.14	-	-
Economic Worry	2.42	1.03	3.22	0.18
Health Worry	1.33	0.68	-	-
Depression	4.28	3.89	3.86	3.98
Anxiety	1.85	2.5	1.58	2.27
Stress	5.37	4.24	5.23	4.79

*- Represent measures that were not measured in the specific phase.*

**TABLE 4:** Correlation table for the variables examined in phase 1.

Variables	Depression	Anxiety	Stress	Age	Economic worry	Health worry	Social disconnectedness	Physical separation	Loneliness	IU
Depression	1									
Anxiety	0.565**									
Stress	0.731**	0.628**								
Age	−0.271**	−0.156**	−0.295**							
Economic stress	0.273**	0.321**	0.287**	−0.173**						
Health worry	0.144**	0.244**	0.096*	0.074	0.193**					
Social Disconnectedness	0.167**	0.113**	0.131**	−0.180**	0.044	0.044				
Physical Separation	0.218**	0.086*	0.077	−0.135**	0.022	0.001	0			
Loneliness	0.545**	0.333**	0.403**	−0.148**	0.150**	0.107*	0.381**	0.208**		
IU	0.419**	0.409**	0.403**	−0.141**	0.230**	0.116**	0.132**	0.065	0.292**	
Gender	0.062	0.062	0.062	0.097*	−0.220**	0.108*	−0.046	0.039	0.093*	0.058

*p < 0.05; **p < 0.01. IU, intolerance of uncertainty. Male gender was coded as 0 and female gender was coded as 1.

At phase 1, the means and SDs for the DASS-21 sub-scales pointed to higher average levels of depression (*M* = 4.28, *SD* = 3.89) and stress (*M* = 5.37, *SD* = 4.24) than reported in the normative data. Anxiety levels (*M* = 1.85, *SD* = 2.5) were similar to those previously reported (Henry and Crawford, 2005).

The average loneliness score (*M* = 35.68, *SD* = 10.22) was also similar to the levels reported in previous population studies (Russell, 1996; Russell et al., 1980). However, the average IU score (*M* = 32.63, *SD* = 9.14) was higher than the scores found among undergraduates and community samples (but was still lower than average scores found in psychiatric samples, such as general anxiety disorder patients (GAD) and obsessive-compulsive (OCD) patients) (Carleton et al., 2012).

#### Predicting Depression

As a first stage, before analyzing the hypothesized model, we created a basic model in which all of the variables were entered as predictors with equal contribution (i.e., all variables were entered in one level for predicting depression). As this model does not account for the significant intercorrelations between the predictive variables, it was indeed found to be non-significant and poorly fitted to the data, as the fit indices suggest (χ^2^_(__100, *N* = 551)_ = 2,870, *p* < 0.001, RMSEA = 0.23, NFI = 0.59, CFI = 0.59). Moreover, a comparison of this basic model with the more theoretical model containing three levels for predicting depression did not reveal a significant difference (ΔChi-square = 32, Δdf = 12, *p* = 0.014). Thus the second model is preferable, due both to the greater number of degrees of freedom and to the fact that it is more theoretically driven.

The results of the SEM model predicting depression with path coefficients (i.e., regression standardized weights) are shown in Figure 2. Gender was not correlated with depression levels and thus was not entered into the model. As predicted by *H1*, social disconnectedness (*β* = 0.36, *p* < 0.001) and physical isolation (*β* = 0.16, *p* < 0.001) were significantly predictive of loneliness levels (*R*^2^ = 0.24). This finding replicates previous research that found a modest correlation between aspects of disconnectedness and perceived loneliness (Hawkley et al., 2003; Cornwell and Waite, 2009). In turn, loneliness was the strongest predictor of depression levels, with a path coefficient of.42 (*p* < 0.001) indicating a moderate-sized effect. Yet, of the two social isolation variables, only physical isolation significantly predicted depression levels (*β* = 0.14, *p* < 0.001), whereas social disconnectedness did not.

Intolerance of uncertainty had a direct effect on depression levels (*β* = 0.24, *p* < 0.001). IU also predicted loneliness levels (*β* = 0.23, *p* < *0.001*), health worry (*β* = 0.13, *p* = *0.002*) and economic worry (*β* = 0.21, *p* < *0.001*). In turn, economic worry (*β* = 0.1, *p* = 0.001) significantly predicted depression levels, and health worry predicted depression levels in a marginally significant manner (*β* = 0.06, *p* = 0.074). Age had a negative direct effect on depression levels (*β* = −0.16, *p* < 0.001), as well as small indirect effects through connections with health worry and economic worry. Younger age predicted higher levels of economic worry (*β* = 0.09, *p* = 0.031), while older age predicted higher levels of health worry (*β* = 0.09, *p* = 0.031).

Factor loadings for the construct of angry evaluation bias ranged from 0.67 to 0.92, and factor loadings for happy evaluation bias ranged from 0.61 to 0.91. Loadings in these ranges indicated that the constructs were relatively consistent over the different facial emotion intensity levels. Evaluating angry faces more negatively (*β* = −0.21, *p* = 0.015) and happy faces less positively (*β* = 0.23, *p* = 0.006) predicted higher levels of stress due to personal economic state. Furthermore, as predicted, angry evaluation bias significantly predicted depression levels (*β* = 0.07, *p* = *0.031*).

Although performance in the facial evaluation task significantly predicted depression, overall, the first model fit was poor, χ^2^_(__112, *N* = 551)_ = 2,902, *p* < 0.001, RMSEA = 0.21, NFI = 0.59, CFI = 0.6. Therefore, we eliminated these contrasts from the model and examined the fit of this alternative model. The second model was significantly better than the model that contained the facial evaluation biases (Δ*Chi-square* = 2,870, Δ*df* = 103, *p* < 0.001). Indeed, this preferable model is much more parsimonious and most fit indices were well within expected guidelines. The chi-square value was still significant χ^2^_(__9, *N* = 551)_ = 31.7, *p* < 0.001but this result is probably because of the large sample size. Other fit indices suggest that the model provided a close fit to the data; RMSEA = 0.067; NFI = 0.95; CFI = 0.96. Overall, this final model explained 42.4% of the variance in symptoms of depression during the lockdown.

Models for predicting anxiety and stress levels are given in the Supplementary Materials for comprehension purposes only and are beyond the scope of the current study that focuses on depression.

### Phase 2

#### Psychological State at Phase 2

Table 3 lists the means and SDs for the psychological measures in phase 2. Correlations for all the measures of phase 2 are given in Table 5.

**TABLE 5:** Correlation table for variables examined in phase 2.

	Phase 1					Phase 2		
Variables	Depression	Anxiety	Stress	Loneliness	Intolerance of uncertainty	Age	Depression	Anxiety
**Phase 1**								
Depression	1							
Anxiety	0.629**	-						
Stress	0.760**	0.696**	-					
Loneliness	0.514**	0.382**	0.469**	-				
IU	0.410**	0.386**	0.356**	0.186*	-			
Age	−0.386**	−0.219*	−0.454**	-0.172	−0.266**	-		
**Phase 2**								
Depression	0.667**	0.555**	0.565**	0.440**	0.292**	−0.368**	-	
Anxiety	0.428**	0.601**	0.447**	0.291**	0.184*	−0.237**	0.674**	-
Stress	0.528**	0.614**	0.618**	0.364**	0.329**	−0.395**	0.833**	0.701**

*p < 0.05; **p < 0.01. IU, intolerance of uncertainty.

To indicate whether the two samples were equivalent in key variables such as DASS scores and economic and health worry, non-parametric Levene’s tests were performed. Non-parametric Levene’s test showed that the variance for the total DASS score in phase 1 was equal to the variance of phase 2 (*F*_(__678_,_1__)_ = 3.394, *p* = 0.066), and this was also found for the three DASS subscales: Depression (*F*_(__678_,_1__)_ = 2.135, *p* = 0.144); Stress (*F*_(__678_,_1__)_ = 2.639, *p* = 0.105); Anxiety (*F*_(__678_,_1__)_ = 0.034, *p* = 0.853). Furthermore, homogeneity of variance was found for economic worry (*F*_(__678_,_1__)_ = 1.693, *p* = 0.194) as well, but a trend for heterogeneity emerged for health worry (*F*_(__678_,_1__)_ = 3.735, *p* = 0.054). In another analysis using independent *t*-tests and non-parametric Levene’s tests, mean scores and variance of each of the key variables of phase 1 were compared between participants who continued to phase 2 and participants who took part only in phase 1. No difference was found between the two samples in depression, anxiety, stress and total DASS scores, loneliness, physical isolation, social disconnectedness, health-related worry, economic-related worry and intolerance of uncertainty (i.e., for all comparisons, significant levels are *p* > 0.1, except for the health-related worry *F*_(__549_,_1__)_ = 3.22, *p* = 0.073). Thus, the second phase can be considered as representative of the whole sample in phase 1.

In order to examine whether psychological state (in terms of depression, anxiety, and stress) as well as economic worry have changed 5 weeks after the lockdown period, mean scores were compared between phases for the participants who participated in phase 2. The results show a decrease in general distress (i.e., total DASS scores) (*t*_(__128__)_ = 2.79, *p* = 0.006) due to a decrease in depression levels from phase 1 to phase 2 (*t*_(__128__)_ = 2.38, *p* = 0.018), while there was no difference in anxiety and stress levels between the two phases (all *p*s > 0.1). However, an increase in economic worry levels (*t*_(__126__)_ = −5.61, *p* < 0.001) was found.

#### Predicting Depression

**FIGURE 3:**
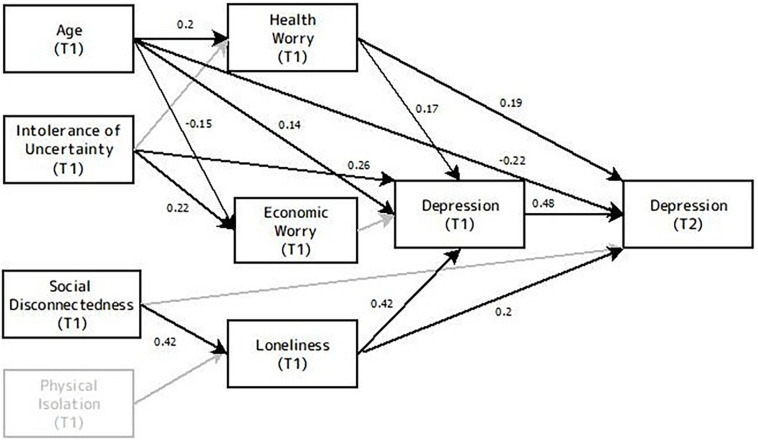
Model of the relationships among age, economic worry, health worry, physical isolation, social disconnectedness, loneliness, intolerance of uncertainty, depression levels from phase 1 and symptoms of depression in phase 2. T1 indicates variables from phase 1, and T2 indicates variables from phase 2. Black lines represent connections in the best fitted model. Gray lines represent non-significant connections that are not included in the final model. Values embedded in unidirectional arrows are standardized regression weights.

Figure 3 shows the results of the SEM model predicting depression in phase 2. The model for phase 2 was based on the research hypotheses and the results found at phase 1. In this model, to control for initial depression levels and to test the continuity of depression symptoms over time, depression levels from phase 1 were also entered. As a consequence of the relatively small number of participants (*N* = 129), the variables of angry and happy evaluation biases were not included in the model.

Our initial model produced a good fit to the data: χ^2^_(__11, *N* = 129)_ = 13.9, *p* = 0.23; RMSEA = 0.046; NFI = 0.94; CFI = 0.98. However, in this model, physical isolation was not related to loneliness levels and did not predict depression levels in phase 2 and thus was excluded from the final model. Moreover, IU did not predict loneliness and health worry, social disconnection did not predict depression levels of phase 1 and economic worry did not predict depression levels in phase 2, so these connections were trimmed from the model as well. The final model was not significantly better than the initial model that contained the connections described above (Δ*Chi-square* = 1.772, Δ*df* = 1, *p* = 0.183), but due to the larger number of degrees of freedom a more parsimonious model is preferable.

Overall, the final model explained 48.2% of the variance in symptoms of depression 5 weeks after the end of the lockdown. The fit indices suggested the model provided an excellent fit to the data. The chi-square value was not significant χ^2^_(__12, *N* = 129)_ = 12.23, *p* = 0.43; RMSEA = 0.012; NFI = 0.95; CFI = 0.99.

As expected, depression levels at phase 1 were the strongest predictors of depression levels 5 weeks after the end of the lockdown, with a path coefficient of 0.48 (*p* < 0.001). However, loneliness at phase 1 remained a strong predictor of depression at phase 2 with a path coefficient of *β* = 0.2 (*p* = 0.009), and the two social isolation variables did not predict depression levels. Age was also predictive of depression levels, with a direct negative effect of *β* = −0.22 (*p* = 0.002), as well as an indirect effect through connection with health worry (*β* = 0.19, *p* = 0.004).

Models for predicting anxiety and stress levels in phase 2 are beyond the scope of the present study and are given in the Supplementary Materials (please see Supplementary Figures S1, S2).

## Discussion

The COVID-19 outbreak is an ongoing global crisis that presents unexpected challenges in all aspects of life. The pandemic had an impact on various psychopathologies, and previous evidence highlighted the risk of future development of depressive symptoms (Torales et al., 2020). Hence, in the current study we explored the influence of subjective loneliness, IU and biased emotional evaluation as well as lockdown-related stress and demographic factors on the non-clinical levels of depressive symptoms during and following the COVID-19 lockdown. Our results show that loneliness, economic worry and young age predict non-clinical depression levels during and following the lockdown. Therefore, our findings call for a psychological-risk-focused policy as a prevention strategy. In a more general perspective, these findings provide another evidence for the influence of subjective loneliness on future development of depressive symptoms.

The results show that levels of non-clinical depression during the lockdown were best predicted by subjective loneliness, as also demonstrated by other studies conducted during the COVID-19 outbreak, both worldwide and in Israel (Killgore et al., 2020; Palgi et al., 2020; Lipskaya-Velikovsky, 2021; Valiente et al., 2021). Furthermore, subjective loneliness predicted depression levels for the short term of 5 weeks after the lockdown, even after controlling for depression levels at the lockdown itself. We also examined the direct effects of *objective isolation* levels and showed that physical isolation and social disconnectedness modestly predicted loneliness during the lockdown, but were not predictive of non-clinical depressive symptoms 5 weeks later. The results further show that during the lockdown individuals felt high levels of personal distress, as indicated by elevated levels of non-clinical depression that persisted even after most of the social distancing restrictions were lifted. Taken together, these results emphasize the importance of relying on subjective measures in predicting depressive symptoms rather than merely on demographic variables. This conclusion is in line with theoretical models that emphasize loneliness as a risk factor for difficulties in emotional and cognitive processes, as researchers have claimed that perceived social isolation is linked to feeling unsafe and creates implicit hypervigilance toward social threat in the environment (Dill and Anderson, 1999; Hawkley and Cacioppo, 2010). One consequence of this maladaptive view of the social world is a diminished capacity for self-regulation, which leads to emotional problems such as stress, pessimism, depression, anxiety and low self-esteem (Cacioppo et al., 2006). Furthermore, our results correspond with previous evidence of a link between loneliness and depression (Hagerty and Williams, 1999; Erzen and Çikrikci, 2018) in different populations including older adults (Gonyea et al., 2018), immigrants (Wu and Penning, 2015), adults who live alone (Park et al., 2017), and adolescents (Lasgaard et al., 2011). Hence, our findings may be generalized to other instances characterized by social isolation. Future studies can use the methodology presented in this study to expand the knowledge regarding the psychological effects of social isolation and loneliness.

Our results further emphasize the impact of *age*. In our sample, age played a significant role in predicting loneliness and depressive symptoms in both phases, such that younger rather than older age predicted more severe levels of non-clinical depression. Older adults are usually considered to be more vulnerable during public health emergency crises (Kar, 2016), but our results indicate that the younger population faces a greater risk in terms of psychological health. In addition to depression, the rapid shutdowns and lockdowns in dozens of countries also affected the economic situation. In our experiment, this effect emerged as a rise in *economic-related worry* from phase 1 to phase 2, with younger age as a predictor of higher levels of economic worry. Furthermore, economic worry was also correlated with non-clinical depression levels at the time of the lockdown, as higher levels of economic worry were related to more depression symptoms. These results correspond with previous studies conducted in Israel during the COVID-19 pandemic, in which age was found to be negatively correlated with anxiety, financial worries, stress and depression symptoms (Horesh et al., 2020; Palgi et al., 2020; Lipskaya-Velikovsky, 2021). Studies from other countries also show a correlation between younger age and elevated levels of emotional distress, such as in Spain (Valiente et al., 2021), Turkey (Ustun, 2020), the United States (Bruine de Bruin, 2021), and the United Kingdom (Shevlin et al., 2020). Another study from Cyprus also found that younger age was connected to lower quality of life, increased anxiety and depression symptoms, and lower levels of compliance with precautionary measures, which may cause higher infection rates among the young adult population (Solomou and Constantinidou, 2020). It is also worth mentioning that a few of these studies also point to female gender as a contributing factor for depression symptoms, though this was not found in the present study. Future studies should further examine the association between gender and depression symptoms. Taken together, the results emphasize that older adults appeared to be more resilient and to have better mental health during the early stages of the pandemic, which is consistent with findings showing better emotion-regulation ability associated with age (Urry and Gross, 2010). These accumulating results highlight the need for specialized interventions for young adults in order to prevent the negative emotional and health-related outcomes of the COVID-19 crisis among this age group.

The lockdown period has also led to *ambiguity and uncertainty* in all aspects of life. Uncertainty itself may be considered threatening (Epstein, 1972), but some individuals may find it more difficult than others to deal with ambiguity and change. Studies have found IU to be a specific risk factor for anxiety, depression and suicide (Carleton et al., 2012). In the present study, IU directly predicted higher levels of non-clinical depression during the lockdown period but not 5 weeks. These findings further contribute to the present literature regarding the influences of IU during the COVID-19 outbreak as it is demonstrating its negative effects on emotional state, as well as its impact on perceived situation-related stress.

So far, most studies examining the psychological effects of the lockdown used explicit and self-report measures. In addition to questionnaires, the present study also used a behavioral task that measured whether biases in the evaluation of expressions of anger and happiness can predict depressive symptoms. As found in previous studies, angry evaluation bias was connected to higher depression levels (Bourke et al., 2010). Furthermore, evaluating angry faces more negatively and happy faces less positively was correlated with higher levels of stress due to personal economic situation. These results suggest that *perceiving facial expressions more negatively than they really are* can predict subjective negative feelings and maintain or reinforce psychological distress. Despite the use of this innovative measuring method, the small number of participants in the second phase precluded the possibility of examining the ability of task performance during the lockdown period to predict psychological measures 5 weeks later. Since cognitive tasks may be a supporting diagnostic tool for psychological distress, future research should evaluate tasks whose value for predicting depression symptoms is stronger.

The present study focused on depression, yet the DASS-21 questionnaire used in this study facilitated further examination of the psychological factors that predicted stress and anxiety after the first lockdown in Israel. For the results of those models, see Supplementary Materials. Health-related worries, economic-related worries and younger age significantly predicted stress, anxiety, and depression. In contrast, level of loneliness solely predicted depression development and was related to stress and anxiety levels only in the first phase. These results further emphasize the unique contribution of loneliness to depression, in line with previous evidence (Cacioppo et al., 2010; Erzen and Çikrikci, 2018; although see Barg et al., 2006; Muyan et al., 2016 for evidence of an association between loneliness and anxiety). Furthermore, the similarities and differences between the predicting factors suggest that despite the comorbidity of depression, anxiety and stress, and their predictors, there are still important differences that should be considered when characterizing individuals at risk and planning individually tailored prevention methods (Richter et al., 2020; for elaboration on individually tailored treatments, see Shani et al., 2019).

This study has several limitations. First, the study was conducted in a complicated time, and therefore ran online based on voluntary participation following adds posted on social medias. This recruitment method made it possible to reach a significant number of participants in the first phase, but did not allow us to control the number of participants who took part in the second phase, as we were able to reach only those who gave the consents and their contact details (i.e., Approximately 60% of the respondents of the first phase). Second, the norms of the implicit task are based on a student sample (see Naor et al., 2018). Nevertheless, the norms for this task may differ across the lifespan, as studies show that older adults exhibit a positivity bias in memory and attention (Mather and Carstensen, 2005; Carstensen and DeLiema, 2018), as well as amplified evaluation of emotional expression (i.e., evaluating both negative and positive emotional faces as more intense compared to younger adults) (Di Domenico et al., 2015). Third, although we created the social isolation questions based on the literature (see Cornwell and Waite, 2009), the questions are limited in their scope as the assessment of the number of social interactions was mainly restricted to activities comprising physical contact and as we were unable to differentiate between different causes for current employment status. Future studies should use a broader set of questions that use other means of communication (e.g., use of social media) to examine objective social isolation status during a quarantine. Finally, although we measured participants’ psychological state at two time points, it is possible that additional symptoms developed only later. Future research should examine the development and persistence of depressive symptoms after longer periods to substantiate the findings of this study.

To conclude, this research examined the ongoing influence of the lockdown on non-clinical depression and on other psychological variables. Loneliness during the lockdown was the strongest predictor of depressive symptoms even after the lockdown was over. Age, depression levels and health-related worry at the lockdown, were also predictors of non-clinical depression levels 5 weeks after. These findings call for a shift from current health-related policies that focus on risk groups in terms of physical health and demographic measures only to policies that consider psychological factors as well. Consideration of these factors may enhance the ability to prevent the negative outcomes of a lockdown by creating a formal policy and improving ways of detecting individuals at risk.

## Data Availability Statement

The raw data supporting the conclusions of this article are available at the project’s OSF at the following link: https://osf.io/a72wg/?view_only=cf8d4ce87a7047069d2561b41818b781 and can also be sent by request.

## Ethics Statement

The studies involving human participants were reviewed and approved by University of Haifa Ethics Committee – approval number 141-20. The patients/participants provided their written informed consent to participate in this study.

## Author Contributions

All authors contributed to the idea generation and to the study design. EG and GM performed the data collection and drafted the manuscript. EG and GM performed the data analysis and interpretation of data under the supervision of HO-S. HO-S provided critical revisions. All authors approved the final version of the manuscript for submission.

## Conflict of Interest

The authors declare that the research was conducted in the absence of any commercial or financial relationships that could be construed as a potential conflict of interest.
