# Supplementary material for: Identifying Variables That Predict Depression Following the General Lockdown During the COVID-19 Pandemic
Source: Front Psychol. 2021 May 17;12:680768. doi: 10.3389/fpsyg.2021.680768 (PMC8165248; doi:10.3389/fpsyg.2021.680768)
Supplement: Supplementary file 1 [file Data_Sheet_1.docx]

# Supplementary Materials

## Phase 1

## Predicting Anxiety

To examine the factors predicting anxiety levels during the lockdown period, we constructed a model of the relationships among age, economic worry, health worry, physical isolation, social disconnectedness, loneliness, IU and symptoms of anxiety. The total model explained 27% of the variance in anxiety levels. This model fit the sample data reasonably well, as indicated by the selected overall goodness-of-fit statistics: χ^2^_(11, N = 551)_ = 32.42, *p* =.001; RMSEA = .06; NFI = .93; CFI = .95.

IU was the strongest predictor of anxiety levels, with a path coefficient of .29 (*p*<.001). Economic worry (*β* = .2*, p*<.001) and health worry (*β* = .15*, p<.*001) were both predictors of anxiety levels.

In contrast to the model predicting depression, none of the objective isolation measures (e.g., physical isolation and social disconnectedness) or age were predictive of anxiety levels. Yet, loneliness significantly predicted anxiety levels (*β = .*21*, p*<.001).

## Predicting Stress

To examine the factors predicting stress levels during the quarantine period, we constructed a model of the relationships among age, economic worry, health worry, physical isolation, social disconnectedness, loneliness, intolerance of uncertainty and symptoms of anxiety. The total model explained 31% of the variance in stress levels. This model fit the sample data well, as indicated by the selected overall goodness-of-fit statistics: χ^2^_(6, N = 551)_ = 8.41, *p* =.209; RMSEA = .026, NFI = .98, CFI = .99.

Loneliness was the strongest predictor of stress levels, with a path coefficient of .28 (*p*<.001). None of the objective isolation measures was predictive of stress levels. Economic worry (*β* = .15*, p*<.001) and IU (*β*=.26*, p*<.001) positively predicted stress levels. Age negatively predicted stress levels (*β=*-.19, *p<.*001). Health worry was not significantly correlated with stress levels (*β =* .02*, p=.*52), so it was removed from the final model.

## Phase 2

## Predicting Anxiety

To examine the factors predicting anxiety levels five weeks after the lockdown period, we constructed a model of the relationships among age, economic worry, health worry, physical isolation, social disconnectedness, loneliness, IU, anxiety levels as phase 1 and symptoms of anxiety at phase 2 (see Figure S1). As in the model of depression, physical isolation did not predict anxiety levels in phase 2 and thus was excluded from the final model. Moreover, IU did not predict loneliness, health worry and anxiety at phase 2, social disconnectedness did not predict anxiety levels, and loneliness did not predict anxiety at phase 2, so these connections were eliminated as well. This final model was not significantly better than the initial model (*ΔChi-square* =4.13, *Δdf*= 3, *p*=.25), but is preferable due to the larger number of degrees of freedom.

The final model explained 36.3% of the variance in anxiety levels in the second phase. This model fit the sample data well, as indicated by the selected overall goodness-of-fit statistics: χ^2^_(14,_ *_N_* _= 129)_ = 18.08, *p* =.2; RMSEA=.048; NFI =.9; CFI =.97.

** Figure 1 is about here **

Anxiety levels from phase 1 was the strongest predictor of anxiety in the second phase, with a path coefficient of .51 (*p*<.001). Health worry from phase 1 was also a predictor for anxiety levels as phase 2 (*β* =.29*, p*=.023), and age marginally negatively predicted anxiety levels directly (*β*=-.14*, p*=.059) and through economic worry (*β* =.12*, p*=.09). Loneliness (*β=*.31*, p*<.001) and IU (*β* =.33*, p*<.001) significantly predicted anxiety levels only at phase 1, thus contributing to general anxiety levels at phase 2 only indirectly.

## Predicting Stress

To examine the factors predicting stress levels five weeks after the lockdown period, we constructed a model of the relationships among age, economic worry, health worry, physical isolation, social disconnectedness, loneliness, IU and symptoms of stress at phase 1 to predict stress levels at phase 2 (see Figure S2). As in the model of depression, physical isolation and social disconnectedness did not predict stress levels in phase 2 and thus were excluded from the final model. Furthermore, the direct connections between loneliness and IU to stress at phase 2 were not significant and were excluded from the final model as well. This final model was not significantly better than the initial model (*ΔChi-square* =2.2, *Δdf*= 3, *p*=.58), but is preferable due to the larger number of degrees of freedom.

The final model explained 45.1% of the variance in stress levels. This model fit the sample data poorly, as indicated by the goodness-of-fit statistics: χ^2^_(14,_ *_N_* _= 129)_ = 15.72, *p* =.33; RMSEA=.031; NFI = .93; CFI = .99.

** Figure 2 is about here **

Stress levels from phase 1 was the strongest predictor of stress in the second phase, with a path coefficient of .48 (*p*<.001). Health worry from phase 1 was also a strong predictor for stress levels as phase 2 (*β* =.29*, p*<.001), and age also negatively predicted stress levels directly (*β*=-.24 *, p*=.004) and through economic worry (*β* = .14*, p*=.036) and health worry. Loneliness (*β =*.38*, p*<.001) and IU (*β* = .2*, p*=.006) significantly predicted stress levels only at phase 1, thus contributing to general stress levels at phase 2 only indirectly.

**Figures Captions.**

**Figure S1.** Model of the relationships among age, economic worry, health worry, social disconnectedness, loneliness, intolerance of uncertainty, and symptoms of anxiety in phase 2. Rectangles represent observed variables. T1 indicates variables from phase 1, and T2 indicates variables from phase 2. Values embedded in unidirectional arrows are standardized regression weights.

**Figure S2.** Model of the relationships among age, economic worry, health worry, social disconnectedness, loneliness, intolerance of uncertainty, and stress in phase 2. Rectangles represent observed variables. T1 indicates variables from phase 1, and T2 indicates variables from phase 2. Values embedded in unidirectional arrows are standardized regression weights.
