# Supplementary figures and images for: Identifying Variables That Predict Depression Following the General Lockdown During the COVID-19 Pandemic
Source: Front Psychol. 2021 May 17;12:680768. doi: 10.3389/fpsyg.2021.680768 (PMC8165248; doi:10.3389/fpsyg.2021.680768)

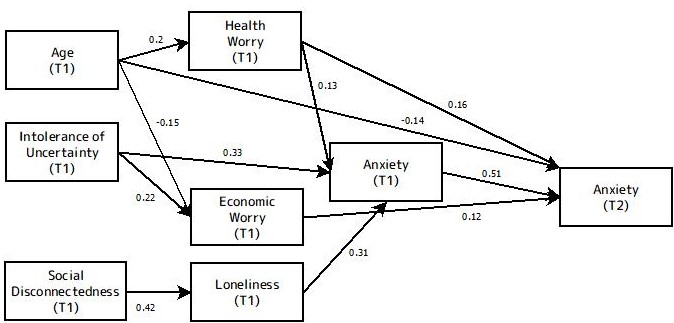

Supplement: Supplementary file 2 [file Image_1.JPEG]

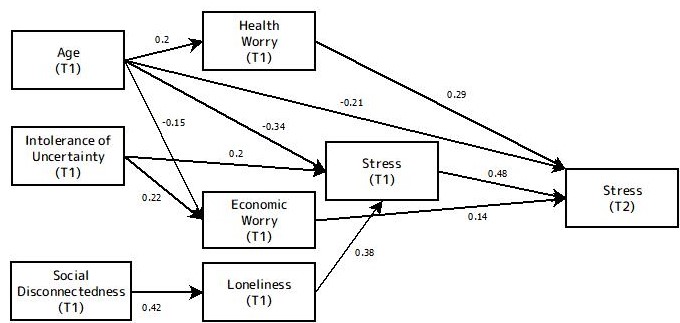

Supplement: Supplementary file 3 [file Image_2.JPEG]
